# A mindfulness-based, stress and coping model of craving in methamphetamine users

**DOI:** 10.1371/journal.pone.0249489

**Published:** 2021-05-18

**Authors:** Massy Mutumba, Judith T. Moskowitz, Torsten B. Neilands, Ji-Young Lee, Samantha E. Dilworth, Adam W. Carrico

**Affiliations:** 1 Department of Health Behavior and Biological Sciences, University of Michigan, Ann Arbor, Michigan, United States of America; 2 Department of Medical Social Sciences, Northwestern University, Evanston, Illinois, United States of America; 3 School of Medicine, University of California San Francisco, San Francisco, California, United States of America; 4 School of Medicine, University of Miami, Coral Gables, Florida, United States of America; University of Queensland, AUSTRALIA

## Abstract

There is increasing interest in the role of mindfulness and mindfulness-based interventions to optimize recovery from a substance use disorder (SUD). However, relatively little is known about the theory-based psychological and social pathways whereby mindfulness could have beneficial effects for managing a chronic, relapsing SUD. Informed by Revised Stress and Coping Theory, the present cross-sectional study examined affective, cognitive, and social pathways whereby mindfulness is associated with lower methamphetamine craving. A total of 161 HIV-positive, methamphetamine-using sexual minority men completed a screening visit for a randomized controlled trial. Using a hybrid structural equation model, we examined pathways whereby mindfulness is associated with lower methamphetamine craving. We found that greater mindfulness was directly associated with lower negative affect and higher positive affect as well as indirectly associated with less methamphetamine craving. Interestingly, the indirect association between mindfulness and methamphetamine craving appeared to be uniquely attributable to positive affect. Only positive affect was indirectly associated with lower methamphetamine craving via higher positive re-appraisal coping and greater self-efficacy for managing triggers for methamphetamine use. Methamphetamine craving was supported by moderate associations with greater substance use severity and more frequent methamphetamine use. These findings support the role of mindfulness in cultivating positive affect, which could be crucial to build the capacity of individuals to manage methamphetamine craving as a chronic stressor that threatens recovery from SUD.

## Introduction

Substance use disorders (SUD) are one of the most prevalent and costly public health challenges throughout the world. In the United States, approximately one in ten people will meet diagnostic criteria for a SUD in their lifetime [1]. In 2016, 20.1 million people aged 12 or older in the United States had a SUD related to their use of alcohol or other substances in the past year [2]. Of these, 684,000 people aged 12 or older had a methamphetamine use disorder [3]. Although evidence-based treatments such as cognitive-behavioral therapy and motivational interviewing are available for stimulant use disorders [4, 5], less than 15% of people living with a SUD seek formal treatment [3]. Among those who seek treatment, up to 40–70% relapse to substance use [6, 7]. Novel approaches are needed to engage the broader population of people living with a SUD that do not seek formal treatment and optimize the long-term recovery of those receiving SUD treatment.

There is increasing interest in the role of mindfulness and mindfulness-based interventions to optimize recovery from a SUD. Jon Kabat-Zinn, the founder of Mindfulness Based Stress Reduction, described mindfulness as, ‘‘the awareness that emerges through paying attention on purpose, in the present moment, and non-judgmentally to the unfolding of experience moment by moment” [8]. Previous studies have observed that dispositional mindfulness is associated with reduced substance use craving [9], especially in the presence of negative affective states [10, 11]. Prior randomized controlled trials (RCTs) also provide support for the efficacy of mindfulness-based interventions for individuals living with SUD [12–14]. Most recently, we observed that a mindfulness-based positive affect intervention achieved greater reductions in methamphetamine craving and self-reported stimulant use in HIV-positive, methamphetamine-using sexual minority men receiving contingency management [15].

Craving, the subjective experience of an urge or desire to use substances [16], has been conceptualized as an emotional and motivational state resulting from activation in brain networks relevant to attention, interoception, and reward processing [17–19]. The clinical relevance of craving is supported by its addition Diagnostic and Statistical Manual of Mental Disorders–Fifth Edition (DSM-5) as a diagnostic criteria for SUD [20]. Consistent with negative reinforcement models of addiction, substance use may become an over-learned behavioral response to escape negative affect or craving [21–23]. In fact, the benefits of mindfulness-based interventions for people living with a SUD may be most pronounced among those with elevated levels of negative affect [24]. An important gap is that relatively little is known about the affective, cognitive, and social pathways through which mindfulness modulates craving, which is crucial to inform the development of novel intervention approaches for people living with SUD.

Revised Stress and Coping Theory proposes that positive affect has unique adaptive significance in the midst of chronic stress such as managing methamphetamine craving [25]. Positive affect may be crucial to sustaining self-regulation efforts in the midst of chronic stress by re-energizing cognitive-behavioral coping responses and building social support to effectively manage a SUD [26, 27]. Consistent with the Revised Stress and Coping Theory, previous studies have substantiated the role of affective and cognitive mechanisms of mindfulness-based interventions [28–32]. Recent studies have found that positive affect shapes primary appraisals (i.e. perceptions of the stressful event as harmful, threatening, or challenging) and secondary stress appraisals (i.e. evaluation of ability and resources available to cope with the stressor) as well as buffers the effects of negative affect that is associated with substance use [31–33]. Positive affect and related cognitive processes are believed to energize and maintain one another through self-reinforcing dynamics that have been termed as an upward spiral [32, 34, 35]. There is also recognition that positive affect builds social support [36], which is crucial to support recovery from a SUD. Social factors such as supportive communications have been identified as mechanisms of action for mindfulness-based interventions [30], but their role in SUD craving has not been evaluated.

**Fig 1:**
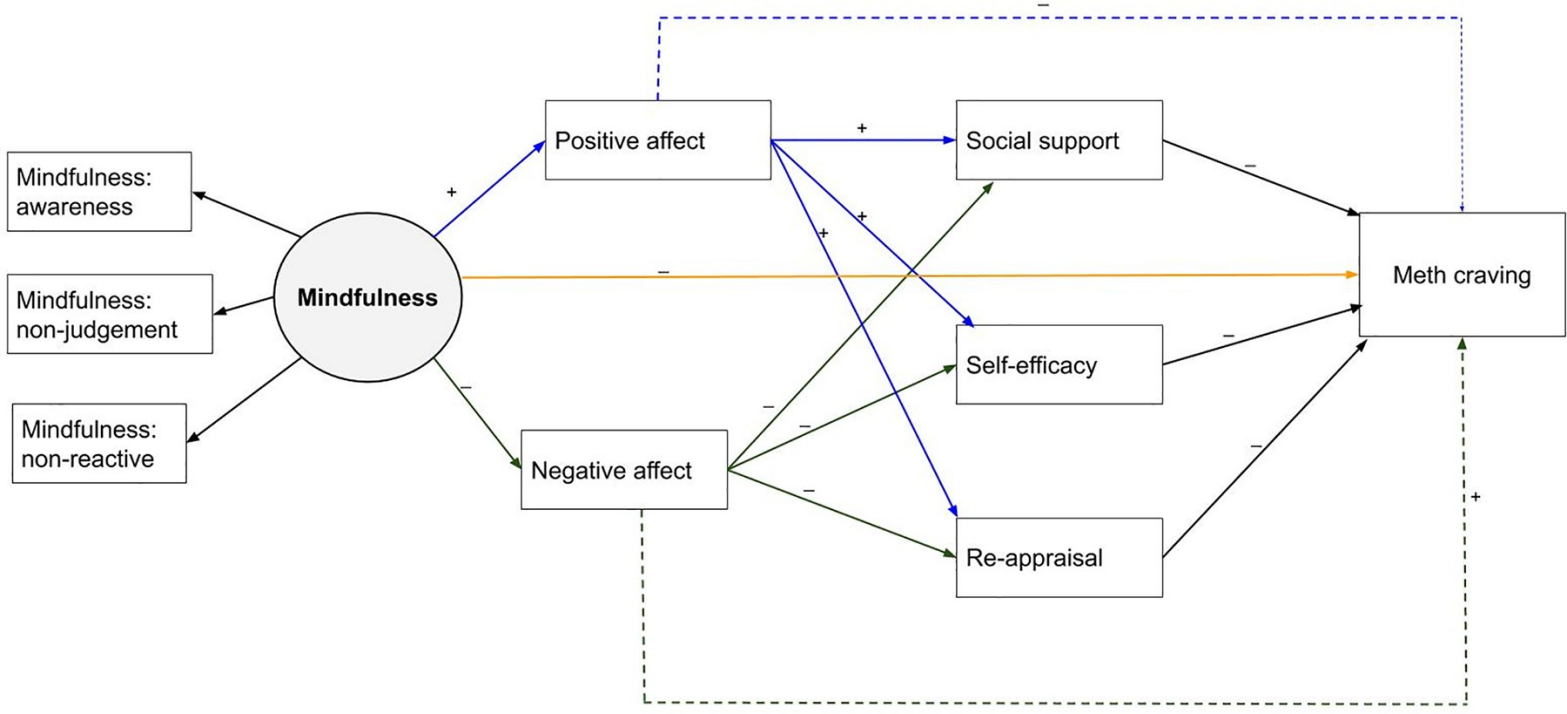
Theoretical pathways linking dispositional mindfulness to methamphetamine craving.

The goal of the present cross-sectional study was to examine theory-based affective, cognitive, and social pathways that could explain the association of mindfulness with lower methamphetamine craving (see Fig 1). Our model conceptualized mindfulness as a trait and affect as a state that mediates the relationship between mindfulness and methamphetamine craving. We hypothesized that mindfulness facilitates metacognitive awareness of affective (i.e. positive and negative affect), cognitive (i.e. self-efficacy and re-appraisal) and social (i.e., social support for abstinence) responses to achieve greater depth of processing in primary and secondary stress appraisals, which in turn leads to more effective management of methamphetamine craving. Consistent with Revised Stress and Coping Theory, we hypothesized that positive affect would uniquely account for the beneficial association of mindfulness with lower methamphetamine craving because it re-invigorates key cognitive (i.e., positive re-appraisal, self-efficacy for managing methamphetamine triggers) and social (i.e., abstinence-specific social support) processes relevant to recovery from a SUD.

## Materials and methods

### Data collection

The present study leveraged data from the screening visit for a randomized controlled trial (RCT) of a mindfulness-based, positive affect intervention for HIV-positive, methamphetamine-using gay, bisexual, and other men who have sex with men (referred to here as sexual minority men), which was conducted in San Francisco, CA USA in collaboration with a community-based program (www.clinicaltrials.gov; NCT01926184). A detailed description of the study protocol is published elsewhere [37, 38]. All relevant procedures were approved by the Institutional Review Boards for the University of California, San Francisco with reliance agreements at the University of Miami and Northwestern University. This RCT received a certificate of confidentiality from the National Institute on Drug Abuse. The University of California, Los Angeles Data Safety and Monitoring Board for Addiction Medicine held annual meetings to review participant-related events and overall progress for this RCT. There were no adverse events or serious adverse events.

### Data collection

Between 2013 and 2017, HIV-positive, methamphetamine-using sexual minority men were recruited through three primary sources. First, men initiating services at a community-based contingency management program completed a brief consent form to be contacted by study staff to learn more about the RCT. Second, direct recruitment was conducted using flyers and palm cards that were distributed in HIV medical clinics, AIDS service organizations, bars and clubs, bath houses, and via social media. Third, an incentivized snowball sampling method was employed where eligible participants received a maximum of $30 for referring up to three individuals who were subsequently judged to be eligible for the RCT. Data for the present study were drawn from the screening visit completed by 161 participants. To be eligible for the screening visit, participants had to meet the following inclusion criteria: 1) 18 years of age or older; 2) identify as a sexual minority man; and 3) provide documentation of HIV-positive serostatus (i.e., letter of diagnosis or ART medications other than Truvada that are matched to their photo identification; and 4) report engaging in methamphetamine use in the past three months.

### Measures

#### Dispositional mindfulness

Dispositional mindfulness was measured using the Five Facet Mindfulness Questionnaire (FFMQ; Baer et al., 2006), a 39-item scale comprising five domains: observing, describing, acting with awareness, non-judging of inner experience, and non-reactivity to inner experience. The present analyses focused on three sub-scales—acting with awareness (“awareness), non-judging of inner experience (“non-judgmental), and non-reactivity to inner experience (“non-reactive”), which have been associated with substance use. Participants rated items on a five-point Likert-type scale (1 = never or very rarely true, 5 = very often or always true), from which a total dispositional mindfulness score was computed. Reliability of each sub-scale used in the present investigation was adequate: awareness (Cronbach’s α = 0.78; M = 24.6, SD = 6.6), non-judgement (Cronbach’s α = 0.87; M = 56.7, SD = 15.1), and non-reactivity (Cronbach’s α = 0.77; M = 44.3, SD = 9.7).

#### Methamphetamine craving

The Penn Alcohol Craving Scale (PACS; Flannery, Volpicelli & Pettinati, 1999), a five-item self-report measure, was adapted to assess craving for methamphetamine. The PACS measures frequency, intensity, and duration of craving as well as an overall rating of craving for the previous week. Reliability of the PACS was adequate (Cronbach’s α = 0.90; M = 2.79, SD = 1.4).

#### Substance use

The Addiction Severity Index (ASI) was administered to assess the severity of alcohol and other substance use [21]. The ASI Drug composite score includes the self-reported number of days using multiple illicit substances during the past 30 days, perceived impairment related to substance use, and perceived need for SUD treatment. Using this scale, participants reported the number of days that they used methamphetamine in the past 30 days as well as how frequently they used methamphetamine in the past three months, with responses measured on a scale from Not at all (0) to daily (7).

#### Positive and negative affect

An adapted version of the Differential Emotions Scale (DES) assessed the frequency of positive and negative affect [39]. The DES was modified to include more items assessing positive affect [40]. Reliability for the 14-item positive affect (Cronbach’s α = 0.89; M = 25.5, SD = 6.9) and the 12-item negative affect (Cronbach’s α = 0.88; M = 13.3, SD = 5.8) subscales of the DES was acceptable.

#### Abstinence-specific social support

The Processes of Change measure for cocaine users was adapted to include strategies that may be employed to avoid methamphetamine use [41]. The six items were drawing from the helping relationships and reinforcement management subscales. These items had adequate internal consistency (Cronbach’s α = 0.90; M = 18.4, SD = 6.4).

#### Self-efficacy

The 8-item brief version of the Situational Confidence Questionnaire (SCQ) was adapted to examine participants’ confidence to resist their urges to use methamphetamine with the original eight SCQ subscales (e.g. pleasant times with others, social pressure, physical discomfort). Items were measured on a scale ranging from 0–100% to measure self-efficacy for managing triggers for methamphetamine. The reliability for the 8-item scale was adequate: (Cronbach’s α = 0.84; M = 460.9, SD = 167.4).

#### Positive re-appraisal

An adapted version of the Ways of Coping questionnaire was administered that included additional items to examine positive re-appraisal coping. The 9-item measure includes items such as “you remind yourself of the good things that came out of the situation”, “you came out of the experience better than you went it”, and “you rediscovered what is important in life.” The reliability for the 9-item scale was adequate: (Cronbach’s α = 0.92; M = 2.69, SD = 0.86).

### Analyses

For hypothesis testing, we used the following multi-stage analytic approach. First, we examined the bivariate associations between methamphetamine craving and substance use as a measure of the validity of our adapted measure of methamphetamine craving. Then, we computed Pearson’s correlations to examine zero-order correlations between methamphetamine craving and the independent variables–mindfulness sub-scales, positive and negative affect, social support, situational confidence, and positive re-appraisal (**Table 1**).

**Table 1:** Descriptive statistics and zero-order correlations between methamphetamine craving and the predictor variables.

	Zero-order correlations							
	1	2	3	4	5	6	7	8
1. Awareness								
2. Non-Reactive	0.27**							
3. Non-Judgement	0.47***	0.29**						
4. Re-Appraisal	0.11	0.28**	0.06					
5. Self-Efficacy	0.13*	0.36***	0.21**	0.28***				
6. Social Support	0.04	0.16*	0.11	0.31***	0.23**			
7. Positive Affect	0.22*	0.40***	0.28***	0.31***	0.37***	0.39***		
8. Negative Affect	-0.39***	-0.28***	-0.48***	-0.12**	-0.19**	-0.17***	-0.36***	
9. Meth Craving	-0.21**	-0.23**	-0.30***	-0.27***	-0.51***	-0.06	-0.29***	0.33***

Notes

***p value < .001

**p value < .05

* p value < .10.

Then, we tested a hybrid structural equation model (**Fig 1**) with full-information maximum likelihood estimation using STATA version 15 to examine the theory-based pathways that could account for the association of mindfulness with methamphetamine craving. This was a hybrid structural equation model because mindfulness was measured as a latent variable and all other variables were treated as observed variables due to the modest sample size Model fit was determined using multiple descriptive indices of model fit: non-significant chi-square (χ2), comparative fit index (CFI) values greater than or equal to 0.95, root mean square error of approximation (RMSEA) values less than or equal to 0.06, and standardized root-mean-square residual (SRMR) values less than 0.08 [42]. We also computed the indirect pathways of the association of mindfulness with methamphetamine craving. The bias-corrected (BC) non-parametric bootstrap based on 5,000 bootstrap replications was used to compute the appropriate asymmetric 95% confidence intervals for the indirect pathways [43]. This strategy was used to ensure sufficient numbers of bootstrap replications at the tails of the distributions where the 2.5th and 97.5th percentiles are estimated. Statistical significance for indirect pathways was determined by the confidence interval excluding zero. In sensitivity analyses, we added frequency of methamphetamine use in the past three months, a proxy indicator of addiction severity, as a mediator in the model.

## Results

### Descriptive summary

The sample consisted of 161 participants, 74% identifying as exclusively gay, 47% non-Hispanic/Latino white, and ranging in age from 22 to 69 years (M = 43.8, SD = 8.9). The average education among the participants was a college or trade school (46%) and the median income was between 5,000–11,999 dollars annually. The majority of participants reported using methamphetamine at least 3–6 times are week (41%) or daily (12%) during the past three months. The most prevalent mode of methamphetamine administration was smoking (77%), followed by injection use methamphetamine (52%), snorting (37%), anal insertions (26%), oral ingestion (23%) and snorting heated methamphetamine (17%). At baseline, 34% were receiving disability benefits and 32% were homeless in the last year.

### Bivariate associations

In the bivariate analyses, greater methamphetamine craving was moderately associated with a higher ASI Drug composite score (r = 0.56; p < 0.001) and greater methamphetamine use in the past 30 days (r = 0.47; p < .001), but modestly associated with frequent methamphetamine use in the past three months (r = 0.32; p < .001). As shown in Table 1, results of the zero-order correlations demonstrated significant, inverse associations between methamphetamine craving with the awareness (r = -0.21, p = 0.008), non-reactive (r = -0.23, p = 0.004), and non-judgmental (r = -0.30, p < 0.001) mindfulness subscales, although the strength of these association was modest. Methamphetamine craving was positively associated with negative affect (r = 0.33, p = < 0.001) and negatively associated with positive affect (r = -0.29, p < 0.001), and the strength of these relationships was modest. For the psychosocial mediators, methamphetamine craving was negatively (and modestly) associated with positive re-appraisal (r = -0.27, p < 0.001) and negatively (and moderately) associated with self-efficacy for managing methamphetamine triggers (r = -0.51, p < 0.001). However, methamphetamine craving was not significantly associated with social support for methamphetamine abstinence (r = -0.01, p = 0.44).

### Direct pathways

**Fig 2:**
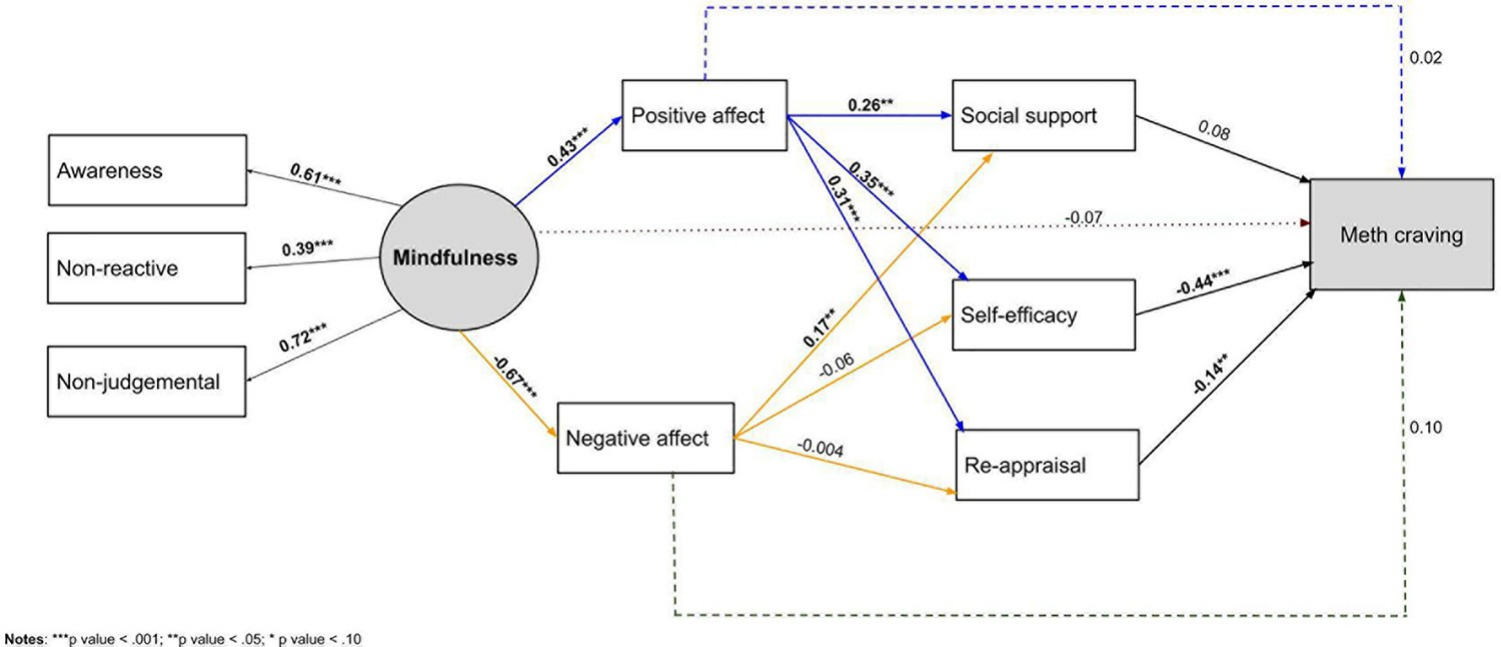
Standardized path analysis coefficients for the model examining the relationship between dispositional mindfulness and methamphetamine craving.

As shown in **Fig 2**, the mindfulness-based, stress and coping model of methamphetamine craving had adequate fit to the data: χ2 (16) = 23.16, p = 0.110; CFI = 0.974; RMSEA = 0.053; SRMR = 0.055. Mindfulness was significantly associated with higher positive affect (B = 0.74; 95% CI = 0.39, 1.1; β = 0.43; p < 0.001) and lower negative affect (B = -0.99; 95% CI = -1.34, -0.64; β = -0.67; p < .001). Positive affect, in turn, was significantly associated with greater social support for abstinence (B = 0.25, 95% CI = 0.09, 0.40; β = 0.26; p = 0.001), self-efficacy for managing methamphetamine triggers (B = 8.65, 95% CI = 4.86, 12.4; β = 0.35; p < .001) and positive re-appraisal coping (B = 0.04; 95% CI = 0.02, 0.06; β = 0.31; p < .001). Negative affect was positively associated with social support for abstinence (B = 0.18; 95% CI = 0.01, 0.36; β = 0.17; p = 0.041), but not significantly associated with self-efficacy for managing methamphetamine triggers and positive re-appraisal coping. Self-efficacy for managing methamphetamine triggers (B = -0.004; 95% CI = -0.01, -0.003; β = -0.44; p < 0.001) and positive re-appraisal coping (B = -0.23; 95% CI = -0.46, -0.001; β = -0.14; p = 0.049) were independently associated with lower methamphetamine craving.

### Indirect pathways

As shown in **Table 2**, the total indirect pathway for the association of mindfulness with methamphetamine craving via positive affect was statistically significant (B = -0.04; 95% CI = -0.05, -0.01; β = -0.17). On the other hand, the total indirect pathway for the association of mindfulness with methamphetamine craving through negative affect was not statistically significant (B = 0.01; 95% CI = -0.03, 0.01; β = 0.04). Of the specific indirect pathways examined, only the pathway from mindfulness through positive affect and self-efficacy was statistically significant: (B = -0.02; 95% CI = -0.04, -0.01).

**Table 2:** Indirect pathways of mindfulness on methamphetamine craving (N = 161).

	*Coef^1^*	*95% CI [BC]^2^*
***Specific indirect pathways***		
Mindfulness -> Positive affect -> Social support -> Methamphetamine craving	0.003	-0.003–0.01
Mindfulness -> Negative affect -> Social support -> Methamphetamine craving	-0.003	-0.01–0.03
Mindfulness -> Positive affect -> Self efficacy -> Methamphetamine craving	-0.02**	-0.04 –-0.01
Mindfulness -> Negative affect -> Self efficacy -> Methamphetamine craving	-0.07	-0.02–0.01
Mindfulness -> Positive affect -> Re-appraisal -> Methamphetamine craving	-0.01	-0.15–0.001
Mindfulness -> Negative affect -> Re-appraisal -> Methamphetamine craving	-0.0001	-0.01–0.01
***Total indirect pathways***		
Mindfulness -> Positive affect -> Mediators -> Methamphetamine craving	-0.03**	-0.05 - -0.01
Mindfulness -> Negative affect -> Mediators -> Methamphetamine craving	-0.01	-0.03–0.01

Notes

***p value < .001

**p value < .05

* p value < .10. Sensitivity analyses.

^1^Unstandardized coefficients.

^2^95% confidence intervals derived from bootstrap analyses.

### Supplemental analyses

As shown in the S1 Fig and S1 Table (appendices), adding frequency of methamphetamine use to the model did not change the estimated direct and indirect path coefficients, or improve the model fit parameters.

## Discussion

Although there is increasing interest in mindfulness-based interventions to optimize the recovery of individuals living with SUD, relatively few studies have examined the mechanisms of action linking mindfulness to substance use craving. To our knowledge, this study is among the first study to examine theory-based affective, cognitive, and social processes linking mindfulness and methamphetamine craving. Consistent with Revised Stress and Coping Theory, greater positive affect emerged as a unique pathway for lower methamphetamine craving through its association with appraisal processes such as self-efficacy and positive re-appraisal coping. Although greater mindfulness was associated with lower negative affect, there was no evidence that negative affect was directly or indirectly associated with lower methamphetamine craving in the multivariate model. Findings highlight the potential benefits of leveraging mindfulness-based approaches to cultivate positive affect to build the capacity of individuals to effectively manage the chronic stressor of methamphetamine craving [15, 44].

The vast majority of prior research has focused on examining the relationship between negative affect and craving, which is consistent with negative reinforcement models of addiction. Previous studies have also shown that dispositional mindfulness is associated with reduced substance use craving [9], especially in the presence of negative affective states [10, 11]. However, the theory-based psychosocial processes mediating the relationship between mindfulness and decreased methamphetamine craving have not been adequately examined [29, 30, 45]. Unlike prior studies [10, 11], negative affect and abstinence-specific social support did not emerge as significant psychosocial mediators [44]. Our study differs in two meaningful ways that could account for these notable differences. First, we included both positive and negative affect in the model, while prior studies have focused exclusively on negative affect. Second, approach to modeling mindfulness as a trait and affect as state also differs from previous studies that have modeled mindfulness as mediating variable [11]. It may be that co-occurrence of positive and negative affect has important consequences for methamphetamine craving. This underscores the importance of differentiating the potentially distinct pathways whereby mindfulness-associated decreases in positive and negative affect could assist individuals with managing craving. As our findings are based on cross-sectional data, there is a need for future research to examine the potentially bidirectional associations among mindfulness, positive and negative affect, and methamphetamine craving in longitudinal studies.

Lastly, we also found that positive affect was independently associated with self-efficacy for managing methamphetamine triggers, social support for abstinence, and positive re-appraisal coping. These findings are consistent with the Revised Stress and Coping Theory and prior research that underscores the unique adaptive significance of positive affect. They are also consistent with results of previous studies indicating the positive affect increases adherence to HIV treatment and HIV viral suppression among stimulant users [46, 47]. Positive affect is thought to sensitize individuals to rewards within their environment and facilitate attainment of desirable outcomes [48]. Positive affect also shapes appraisal processes and individuals who experience positive emotions are more likely to adopt a positive attitude towards the self or others when making evaluative judgments [49, 50]. In addition, positive affect may increase the frequency and intensity of positively valenced cognitions [51] by biasing attention and memory recall toward positive information, promoting positive interpretations of ambiguous situations [32, 49, 52–54], and assigning positive value to objects or thoughts during the cognitive appraisal process [49]. As such, positive affect may be central to counteracting the deleterious effects of negative affect on the cognitive processes associated with craving. Further clinical research testing the efficacy of mindfulness-based, positive affect interventions for supporting recovery from a SUD and optimizing HIV treatment outcomes will assist with elucidating these mechanisms.

Our findings should be interpreted within the limitations of this study. First, our analyses are based on cross-sectional data that could not adequately model the potentially bi-directional associations between psychosocial factors and methamphetamine craving. Feedback loops between negative affect, positive affect, social support, self-efficacy, re-appraisal and methamphetamine craving could affect the directionality and statistical significance of our findings. Additionally, the data were collected using retrospective self-reports which are easily affected by recall bias. Therefore, there is a need for longitudinal analyses to further our understanding of the mechanisms whereby mindfulness could assist with managing craving and reducing substance use. Previous studies have found that the influence of craving on substance use varies with time, and the proximal effects of craving and related moderating factors fluctuates rapidly [55, 56]. Moreover, there is evidence to suggest that single point assessments may not provide an accurate assessment of mindfulness and existing self-report measures cannot accurately capture the central quality of mindfulness [57]. Research methodologies such as the ecological momentary assessment [55, 58] that provide repeated real-time assessments of participants behaviors while in their natural environments could remedy the methodological challenges associated with measuring complex time varying constructs such as craving and mindfulness, to better inform our understanding of the causal relationships between craving, substance use and associated moderating variables. Future longitudinal studies should also examine whether key demographic factors such as gender, different classes of substance use (e.g., opioids), substance use disorder severity, or health status modify the theory-based pathways whereby mindfulness is associated with lower craving. Taken together, our findings underscore the need to examine this model in other substance using populations such as opioid users and with more diverse groups with respect to gender and sexual orientation. Second, participants were recruited in San Francisco, a well-resourced setting with extensive services for HIV-positive methamphetamine-using sexual minority men [59]. Further research is needed to replicate these findings in more representative populations of methamphetamine users.

Despite these limitations, our findings make an important contribution to the burgeoning literature on mindfulness and mindfulness-based interventions for individuals living with a SUD. First, the results provide important insights regarding the theory-based pathways that could link mindfulness with lower methamphetamine craving. These findings could be leveraged to enhance the therapeutic effects of mindfulness-based interventions for individuals living with SUD. Second, the unique adaptive significance of positive affect for reduced methamphetamine craving is consistent with Revised Stress and Coping Theory. Findings will catalyze further clinical research to examine the potential benefits of explicitly leveraging mindfulness-based approaches to cultivate positive affect in people living with SUD.

## Supporting information

S1 Fig Standardized path analysis coefficients for the model examining the relationship between mindfulness and methamphetamine craving, with addition of frequency of methamphetamine use. (TIFF)

S1 Table Indirect pathways of mindfulness on methamphetamine craving, with addition of frequency of methamphetamine use (N = 161). (DOCX)

S1 File (DOCX)

S2 File (DOCX)

S1 Dataset (ZIP)
