# Supplementary material for: A mindfulness-based, stress and coping model of craving in methamphetamine users
Source: PLoS One. 2021 May 18;16(5):e0249489. doi: 10.1371/journal.pone.0249489 (PMC8130914; doi:10.1371/journal.pone.0249489)
Supplement: S1 Table — (DOCX) [file pone.0249489.s002.docx]

**S1 Table. Indirect pathways of mindfulness on methamphetamine craving, with addition of frequency of methamphetamine use (N = 161)**

|  | ***Coef^1^*** | ***95% CI*** |
| --- | --- | --- |
| ***Specific indirect pathways*** | | |
| Mindfulness -> Positive affect -> Social support -> Methamphetamine craving | 0.003 | -0.004 – 0.01 |
| Mindfulness -> Negative affect -> Social support -> Methamphetamine craving | -0.003 | -0.01 – 0.05 |
| Mindfulness -> Positive affect -> Self efficacy -> Methamphetamine craving | -0.03** | -0.05 – -0.01 |
| Mindfulness -> Negative affect -> Self efficacy -> Methamphetamine craving | -0.08 | -0.03 – 0.01 |
| Mindfulness -> Positive affect -> Re-appraisal -> Methamphetamine craving | -0.01 | -0.02 – 0.02 |
| Mindfulness -> Negative affect -> Re-appraisal -> Methamphetamine craving | -0.0002 | -0.01 – 0.01 |
| Mindfulness -> Negative affect -> frequency of methamphetamine use -> Methamphetamine craving | -0.01 | -0.02 – 0.09 |
| Mindfulness -> Positive affect -> frequency of methamphetamine use -> Methamphetamine craving | -0.01 | -0.02 – 0.01 |
| ***Total indirect pathways*** | | |
| Mindfulness -> Positive affect -> Mediators -> Methamphetamine craving | -0.04** | -0.07 - -0.01 |
| Mindfulness -> Negative affect -> Mediators -> Methamphetamine craving | -0.02 | -0.05 – 0.01 |

**Notes:** ^1^Unstandardized coefficients

***p < .001; **p < .05; *p < .10
