# Supplementary material for: A mindfulness-based, stress and coping model of craving in methamphetamine users
Source: PLoS One. 2021 May 18;16(5):e0249489. doi: 10.1371/journal.pone.0249489 (PMC8130914; doi:10.1371/journal.pone.0249489)
Supplement: S1 File — (DOCX) [file pone.0249489.s003.docx]

/*Reporting standardized coeficients*/

sem (Mindfulness -> cent_awareness, ) (Mindfulness -> cent_nonreact, ) (Mindfulness -> cent_nonjudge, )

(Mindfulness -> cent_desposstep, ) (Mindfulness -> cent_desnegstep, ) cent_desposstep -> cent_poc, )

(cent_desposstep -> cent_situ_conf, ) (cent_desposstep -> cent_reappraisal, ) (cent_desposstep -> freqmeth2, )

(cent_desnegstep -> cent_poc, ) (cent_desnegstep -> cent_situ_conf, ) (cent_desnegstep -> cent_reappraisal, )

(cent_desnegstep -> freqmeth2, ) (cent_poc -> cent_pacs, ) (cent_situ_conf -> cent_pacs,)

(cent_reappraisal -> cent_pacs, ) (freqmeth2 -> cent_pacs, ) (Mindfulness -> cent_pacs, )

(cent_desnegstep -> cent_pacs, ) (cent_desposstep -> cent_pacs, ), standardized latent(Mindfulness )

cov(e.cent_poc*e.cent_reappraisal) cov( e.cent_awareness*e.cent_nonreact e.cent_awareness*e.cent_nonjudge e.cent_nonreact*e.cent_desposstep e.cent_nonreact*e.cent_desnegstep e.cent_nonreact*e.cent_situ_conf e.cent_nonreact*e.cent_reappraisal e.cent_nonreact*e.cent_poc e.cent_reappraisal*e.cent_poc) nocapslatent

Endogenous variables

Observed: cent_desposstep cent_desnegstep cent_poc cent_situ_conf cent_reappraisal freqmeth2 cent_pacs

Measurement: cent_awareness cent_nonreact cent_nonjudge

Exogenous variables

Latent: Mindfulness

Fitting target model:

Structural equation model Number of obs = 161

Estimation method = ml

Log likelihood = -4960.2154

( 1) [cent_awareness]Mindfulness = 1

--------------------------------------------------------------------------------------------------------

| OIM

Standardized | Coef. Std. Err. z P>|z| [95% Conf. Interval]

---------------------------------------+----------------------------------------------------------------

Structural |

cent_desposstep |

Mindfulness | .4621163 .0794541 5.82 0.000 .3063891 .6178436

_cons | -1.92e-08 .078811 -0.00 1.000 -.1544668 .1544668

-------------------------------------+----------------------------------------------------------------

cent_desnegstep |

Mindfulness | -.7607813 .0843065 -9.02 0.000 -.9260189 -.5955436

_cons | 2.58e-08 .078811 0.00 1.000 -.1544668 .1544668

-------------------------------------+----------------------------------------------------------------

cent_poc |

cent_desposstep | .2637274 .0791979 3.33 0.001 .1085022 .4189525

cent_desnegstep | .1668087 .0807226 2.07 0.039 .0085952 .3250222

_cons | -4.59e-09 .0761477 -0.00 1.000 -.1492469 .1492468

-------------------------------------+----------------------------------------------------------------

cent_situ_conf |

cent_desposstep | .3509807 .074048 4.74 0.000 .2058492 .4961121

cent_desnegstep | -.0583759 .0782823 -0.75 0.456 -.2118064 .0950547

_cons | -1.11e-08 .0730438 -0.00 1.000 -.1431632 .1431632

-------------------------------------+----------------------------------------------------------------

cent_reappraisal |

cent_desposstep | .3081693 .0769797 4.00 0.000 .1572918 .4590468

cent_desnegstep | -.004378 .0803976 -0.05 0.957 -.1619544 .1531985

_cons | -5.88e-09 .0749353 -0.00 1.000 -.1468706 .1468705

-------------------------------------+----------------------------------------------------------------

freqmeth2 |

cent_desposstep | -.0877034 .0835371 -1.05 0.294 -.251433 .0760263

cent_desnegstep | .072827 .0836247 0.87 0.384 -.0910745 .2367285

_cons | 2.708609 .1699437 15.94 0.000 2.375526 3.041693

-------------------------------------+----------------------------------------------------------------

cent_pacs |

cent_desposstep | .0698898 .084762 0.82 0.410 -.0962407 .2360204

cent_desnegstep | -.0039975 .1736179 -0.02 0.982 -.3442824 .3362874

cent_poc | .0565896 .0666017 0.85 0.396 -.0739472 .1871265

cent_situ_conf | -.4284344 .0638789 -6.71 0.000 -.5536346 -.3032341

cent_reappraisal | -.1283142 .0675417 -1.90 0.057 -.2606936 .0040652

freqmeth2 | .2846949 .0605369 4.70 0.000 .1660447 .4033451

Mindfulness | -.308271 .2172584 -1.42 0.156 -.7340897 .1175477

_cons | -.7711272 .1719201 -4.49 0.000 -1.108085 -.4341699

---------------------------------------+----------------------------------------------------------------

Measurement |

cent_awareness |

Mindfulness | .5055714 .0858915 5.89 0.000 .3372272 .6739156

_cons | 4.49e-10 .078811 0.00 1.000 -.1544668 .1544668

-------------------------------------+----------------------------------------------------------------

cent_nonreact |

Mindfulness | .3904687 .1058823 3.69 0.000 .1829431 .5979942

_cons | -3.35e-09 .078811 -0.00 1.000 -.1544668 .1544668

-------------------------------------+----------------------------------------------------------------

cent_nonjudge |

Mindfulness | .6388314 .0808585 7.90 0.000 .4803515 .7973112

_cons | 3.83e-09 .078811 0.00 1.000 -.1544668 .1544668

---------------------------------------+----------------------------------------------------------------

var(e.cent_awareness)| .7443976 .0868485 .5922371 .9356518

var(e.cent_nonreact)| .8475342 .0826875 .7000225 1.02613

var(e.cent_nonjudge)| .5918945 .1033099 .4204115 .8333242

var(e.cent_desposstep)| .7864485 .0734341 .6549225 .9443884

var(e.cent_desnegstep)| .4212119 .1282776 .2318848 .765119

var(e.cent_poc)| .9335552 .0381938 .8616195 1.011497

var(e.cent_situ_conf)| .8589984 .0507769 .7650261 .9645137

var(e.cent_reappraisal)| .9040639 .0441339 .8215723 .9948381

var(e.freqmeth2)| .9825133 .0204009 .943331 1.023323

var(e.cent_pacs)| .5677248 .0706939 .4447798 .7246541

var(Mindfulness)| 1 . . .

---------------------------------------+----------------------------------------------------------------

cov(e.cent_awareness,e.cent_nonreact)| .0758516 .0807513 0.94 0.348 -.082418 .2341212

cov(e.cent_awareness,e.cent_nonjudge)| .213593 .1057602 2.02 0.043 .0063068 .4208792

cov(e.cent_nonreact,e.cent_desposstep)| .2694357 .0834595 3.23 0.001 .1058581 .4330132

cov(e.cent_nonreact,e.cent_desnegstep)| .0372579 .1314054 0.28 0.777 -.2202919 .2948078

cov(e.cent_nonreact,e.cent_poc)| .0599721 .076452 0.78 0.433 -.0898712 .2098153

cov(e.cent_nonreact,e.cent_situ_conf)| .1981373 .0751652 2.64 0.008 .0508161 .3454584

cov(e.cent_nonreact,e.cent_reappraisal)| .144087 .0757996 1.90 0.057 -.0044775 .2926514

cov(e.cent_poc,e.cent_reappraisal)| .2696895 .0730788 3.69 0.000 .1264576 .4129214

--------------------------------------------------------------------------------------------------------

LR test of model vs. saturated: chi2(17) = 18.22, Prob > chi2 = 0.3752

. estat mindices

Modification indices

------------------------------------------------------------------

| Standard

| MI df P>MI EPC EPC

-------------------+----------------------------------------------

Structural |

cent_poc |

cent_nonreact | 4.390 1 0.04 .4499119 .6755409

-----------------+----------------------------------------------

cent_situ_conf |

cent_nonreact | 5.854 1 0.02 16.06295 .925658

cent_poc | 5.366 1 0.02 4.56293 .1751237

cent_reappraisal | 5.266 1 0.02 34.14988 .1762848

------------------------------------------------------------------

EPC = expected parameter change

. estat gof, stats(all)

----------------------------------------------------------------------------

Fit statistic | Value Description

---------------------+------------------------------------------------------

Likelihood ratio |

chi2_ms(17) | 18.218 model vs. saturated

p > chi2 | 0.375

chi2_bs(45) | 336.657 baseline vs. saturated

p > chi2 | 0.000

---------------------+------------------------------------------------------

Population error |

RMSEA | 0.021 Root mean squared error of approximation

90% CI, lower bound | 0.000

upper bound | 0.076

pclose | 0.747 Probability RMSEA <= 0.05

---------------------+------------------------------------------------------

Information criteria |

AIC | 10016.431 Akaike's information criterion

BIC | 10164.338 Bayesian information criterion

---------------------+------------------------------------------------------

Baseline comparison |

CFI | 0.996 Comparative fit index

TLI | 0.989 Tucker-Lewis index

---------------------+------------------------------------------------------

Size of residuals |

SRMR | 0.044 Standardized root mean squared residual

CD | 0.735 Coefficient of determination

----------------------------------------------------------------------------

**sem, standardized**

Structural equation model Number of obs = 161

Estimation method = ml

Log likelihood = -4960.2154

( 1) [cent_awareness]Mindfulness = 1

--------------------------------------------------------------------------------------------------------

| OIM

Standardized | Coef. Std. Err. z P>|z| [95% Conf. Interval]

---------------------------------------+----------------------------------------------------------------

Structural |

cent_desposstep |

Mindfulness | .4621163 .0794541 5.82 0.000 .3063891 .6178436

_cons | -1.92e-08 .078811 -0.00 1.000 -.1544668 .1544668

-------------------------------------+----------------------------------------------------------------

cent_desnegstep |

Mindfulness | -.7607813 .0843065 -9.02 0.000 -.9260189 -.5955436

_cons | 2.58e-08 .078811 0.00 1.000 -.1544668 .1544668

-------------------------------------+----------------------------------------------------------------

cent_poc |

cent_desposstep | .2637274 .0791979 3.33 0.001 .1085022 .4189525

cent_desnegstep | .1668087 .0807226 2.07 0.039 .0085952 .3250222

_cons | -4.59e-09 .0761477 -0.00 1.000 -.1492469 .1492468

-------------------------------------+----------------------------------------------------------------

cent_situ_conf |

cent_desposstep | .3509807 .074048 4.74 0.000 .2058492 .4961121

cent_desnegstep | -.0583759 .0782823 -0.75 0.456 -.2118064 .0950547

_cons | -1.11e-08 .0730438 -0.00 1.000 -.1431632 .1431632

-------------------------------------+----------------------------------------------------------------

cent_reappraisal |

cent_desposstep | .3081693 .0769797 4.00 0.000 .1572918 .4590468

cent_desnegstep | -.004378 .0803976 -0.05 0.957 -.1619544 .1531985

_cons | -5.88e-09 .0749353 -0.00 1.000 -.1468706 .1468705

-------------------------------------+----------------------------------------------------------------

freqmeth2 |

cent_desposstep | -.0877034 .0835371 -1.05 0.294 -.251433 .0760263

cent_desnegstep | .072827 .0836247 0.87 0.384 -.0910745 .2367285

_cons | 2.708609 .1699437 15.94 0.000 2.375526 3.041693

-------------------------------------+----------------------------------------------------------------

cent_pacs |

cent_desposstep | .0698898 .084762 0.82 0.410 -.0962407 .2360204

cent_desnegstep | -.0039975 .1736179 -0.02 0.982 -.3442824 .3362874

cent_poc | .0565896 .0666017 0.85 0.396 -.0739472 .1871265

cent_situ_conf | -.4284344 .0638789 -6.71 0.000 -.5536346 -.3032341

cent_reappraisal | -.1283142 .0675417 -1.90 0.057 -.2606936 .0040652

freqmeth2 | .2846949 .0605369 4.70 0.000 .1660447 .4033451

Mindfulness | -.308271 .2172584 -1.42 0.156 -.7340897 .1175477

_cons | -.7711272 .1719201 -4.49 0.000 -1.108085 -.4341699

---------------------------------------+----------------------------------------------------------------

Measurement |

cent_awareness |

Mindfulness | .5055714 .0858915 5.89 0.000 .3372272 .6739156

_cons | 4.49e-10 .078811 0.00 1.000 -.1544668 .1544668

-------------------------------------+----------------------------------------------------------------

cent_nonreact |

Mindfulness | .3904687 .1058823 3.69 0.000 .1829431 .5979942

_cons | -3.35e-09 .078811 -0.00 1.000 -.1544668 .1544668

-------------------------------------+----------------------------------------------------------------

cent_nonjudge |

Mindfulness | .6388314 .0808585 7.90 0.000 .4803515 .7973112

_cons | 3.83e-09 .078811 0.00 1.000 -.1544668 .1544668

---------------------------------------+----------------------------------------------------------------

var(e.cent_awareness)| .7443976 .0868485 .5922371 .9356518

var(e.cent_nonreact)| .8475342 .0826875 .7000225 1.02613

var(e.cent_nonjudge)| .5918945 .1033099 .4204115 .8333242

var(e.cent_desposstep)| .7864485 .0734341 .6549225 .9443884

var(e.cent_desnegstep)| .4212119 .1282776 .2318848 .765119

var(e.cent_poc)| .9335552 .0381938 .8616195 1.011497

var(e.cent_situ_conf)| .8589984 .0507769 .7650261 .9645137

var(e.cent_reappraisal)| .9040639 .0441339 .8215723 .9948381

var(e.freqmeth2)| .9825133 .0204009 .943331 1.023323

var(e.cent_pacs)| .5677248 .0706939 .4447798 .7246541

var(Mindfulness)| 1 . . .

---------------------------------------+----------------------------------------------------------------

cov(e.cent_awareness,e.cent_nonreact)| .0758516 .0807513 0.94 0.348 -.082418 .2341212

cov(e.cent_awareness,e.cent_nonjudge)| .213593 .1057602 2.02 0.043 .0063068 .4208792

cov(e.cent_nonreact,e.cent_desposstep)| .2694357 .0834595 3.23 0.001 .1058581 .4330132

cov(e.cent_nonreact,e.cent_desnegstep)| .0372579 .1314054 0.28 0.777 -.2202919 .2948078

cov(e.cent_nonreact,e.cent_poc)| .0599721 .076452 0.78 0.433 -.0898712 .2098153

cov(e.cent_nonreact,e.cent_situ_conf)| .1981373 .0751652 2.64 0.008 .0508161 .3454584

cov(e.cent_nonreact,e.cent_reappraisal)| .144087 .0757996 1.90 0.057 -.0044775 .2926514

cov(e.cent_poc,e.cent_reappraisal)| .2696895 .0730788 3.69 0.000 .1264576 .4129214

--------------------------------------------------------------------------------------------------------

LR test of model vs. saturated: chi2(17) = 18.22, Prob > chi2 = 0.3752
