# Supplementary material for: A mindfulness-based, stress and coping model of craving in methamphetamine users
Source: PLoS One. 2021 May 18;16(5):e0249489. doi: 10.1371/journal.pone.0249489 (PMC8130914; doi:10.1371/journal.pone.0249489)
Supplement: S2 File — (DOCX) [file pone.0249489.s004.docx]

. use "U:\Working papers\ARTEMIS study\Data\BL data- workingdata.dta"

. sem (Mindfulness -> cent_awareness, ) (Mindfulness -> cent_nonreact, ) (Mindfulness -> cent_nonjudge, ) ///

> (Mindfulness -> cent_desposstep, ) (Mindfulness -> cent_desnegstep, ) (cent_desposstep -> cent_poc, ) ///

> (cent_desposstep -> cent_situ_conf, ) (cent_desposstep -> cent_reappraisal, ) (cent_desnegstep -> cent_poc, ) ///

> (cent_desnegstep -> cent_situ_conf, ) (cent_desnegstep -> cent_reappraisal, ) (cent_poc -> cent_pacs, ) ///

> (cent_situ_conf -> cent_pacs,) (cent_reappraisal -> cent_pacs, ) (Mindfulness -> cent_pacs, ) ///

> (cent_desnegstep -> cent_pacs, ) (cent_desposstep -> cent_pacs, ), cov(e.cent_poc*e.cent_reappraisal) ///

> cov(e.cent_nonreact*e.cent_desposstep) cov(e.cent_nonreact*e.cent_situ_conf) latent(Mindfulness ) standardized

Endogenous variables

Observed: cent_desposstep cent_desnegstep cent_poc cent_situ_conf cent_reappraisal cent_pacs

Measurement: cent_awareness cent_nonreact cent_nonjudge

Exogenous variables

Latent: Mindfulness

Fitting target model:

Iteration 0: log likelihood = -4824.9965 (not concave)

Iteration 1: log likelihood = -4808.8774 (not concave)

Iteration 2: log likelihood = -4778.0793 (not concave)

Iteration 3: log likelihood = -4765.5123

Iteration 4: log likelihood = -4764.2758

Iteration 5: log likelihood = -4761.5824

Iteration 6: log likelihood = -4761.3891

Iteration 7: log likelihood = -4761.3881

Iteration 8: log likelihood = -4761.3881

Structural equation model Number of obs = 161

Estimation method = ml

Log likelihood = -4761.3881

( 1) [cent_awareness]Mindfulness = 1

-------------------------------------------------------------------------------------------------------

| OIM

Standardized | Coef. Std. Err. z P>|z| [95% Conf. Interval]

--------------------------------------+----------------------------------------------------------------

Structural |

cent_desposstep |

Mindfulness | .4316191 .0804179 5.37 0.000 .2740029 .5892352

_cons | -5.37e-09 .078811 -0.00 1.000 -.1544668 .1544668

------------------------------------+----------------------------------------------------------------

cent_desnegstep |

Mindfulness | -.6735287 .0666559 -10.10 0.000 -.8041719 -.5428855

_cons | 2.64e-09 .078811 0.00 1.000 -.1544668 .1544668

------------------------------------+----------------------------------------------------------------

cent_poc |

cent_desposstep | .2630245 .0786652 3.34 0.001 .1088436 .4172054

cent_desnegstep | .1663648 .0802513 2.07 0.038 .0090752 .3236545

_cons | -4.33e-09 .0759447 -0.00 1.000 -.1488488 .1488488

------------------------------------+----------------------------------------------------------------

cent_situ_conf |

cent_desposstep | .3507359 .0739116 4.75 0.000 .2058719 .4955999

cent_desnegstep | -.0602825 .0775899 -0.78 0.437 -.2123559 .0917908

_cons | -1.10e-08 .0731313 -0.00 1.000 -.1433347 .1433346

------------------------------------+----------------------------------------------------------------

cent_reappraisal |

cent_desposstep | .3081953 .0768178 4.01 0.000 .1576352 .4587554

cent_desnegstep | -.0043783 .0804113 -0.05 0.957 -.1619816 .1532249

_cons | -6.13e-09 .0749415 -0.00 1.000 -.1468826 .1468826

------------------------------------+----------------------------------------------------------------

cent_pacs |

cent_desposstep | .0226371 .0809549 0.28 0.780 -.1360315 .1813057

cent_desnegstep | .1009671 .1080276 0.93 0.350 -.1107631 .3126972

cent_poc | .083906 .0700693 1.20 0.231 -.0534272 .2212392

cent_situ_conf | -.4370251 .0662433 -6.60 0.000 -.5668595 -.3071907

cent_reappraisal | -.1395111 .0711713 -1.96 0.050 -.2790044 -.0000179

Mindfulness | -.2018547 .1324634 -1.52 0.128 -.4614782 .0577688

_cons | -8.07e-09 .0657776 -0.00 1.000 -.1289217 .1289217

--------------------------------------+----------------------------------------------------------------

Measurement |

cent_awareness |

Mindfulness | .6092498 .0674688 9.03 0.000 .4770134 .7414861

_cons | 6.19e-09 .078811 0.00 1.000 -.1544668 .1544668

------------------------------------+----------------------------------------------------------------

cent_nonreact |

Mindfulness | .3919424 .0812958 4.82 0.000 .2326056 .5512793

_cons | 2.61e-09 .078811 0.00 1.000 -.1544668 .1544668

------------------------------------+----------------------------------------------------------------

cent_nonjudge |

Mindfulness | .7235337 .0633178 11.43 0.000 .5994331 .8476344

_cons | 1.31e-08 .078811 0.00 1.000 -.1544668 .1544668

--------------------------------------+----------------------------------------------------------------

var(e.cent_awareness)| .6288147 .0822107 .4866731 .8124713

var(e.cent_nonreact)| .8463811 .0637266 .7302582 .9809694

var(e.cent_nonjudge)| .4764989 .0916252 .3268781 .6946052

var(e.cent_desposstep)| .813705 .0694198 .6884117 .9618021

var(e.cent_desnegstep)| .5463591 .0897894 .3959046 .7539903

var(e.cent_poc)| .9285825 .0407913 .8519781 1.012075

var(e.cent_situ_conf)| .8610573 .0502034 .7680744 .9652967

var(e.cent_reappraisal)| .9042119 .0441858 .8216274 .9950973

var(e.cent_pacs)| .655852 .062762 .543688 .7911556

var(Mindfulness)| 1 . . .

--------------------------------------+----------------------------------------------------------------

cov(e.cent_nonreact,e.cent_desposstep)| .2744777 .0777877 3.53 0.000 .1220167 .4269388

cov(e.cent_nonreact,e.cent_situ_conf)| .2231382 .0729899 3.06 0.002 .0800806 .3661958

cov(e.cent_poc,e.cent_reappraisal)| .2696886 .073079 3.69 0.000 .1264564 .4129207

-------------------------------------------------------------------------------------------------------

LR test of model vs. saturated: chi2(16) = 23.16, Prob > chi2 = 0.1096

. estat mindices

Modification indices

------------------------------------------------------------------

| Standard

| MI df P>MI EPC EPC

-------------------+----------------------------------------------

Structural |

cent_poc |

cent_pacs | 4.440 1 0.04 -1.67304 -.369786

-----------------+----------------------------------------------

cent_situ_conf |

cent_poc | 4.401 1 0.04 4.02236 .1549748

-----------------+----------------------------------------------

cent_reappraisal |

cent_nonreact | 4.215 1 0.04 .0146082 .1637762

------------------------------------------------------------------

EPC = expected parameter change

. estat gof, stats(all)

----------------------------------------------------------------------------

Fit statistic | Value Description

---------------------+------------------------------------------------------

Likelihood ratio |

chi2_ms(16) | 23.158 model vs. saturated

p > chi2 | 0.110

chi2_bs(36) | 311.759 baseline vs. saturated

p > chi2 | 0.000

---------------------+------------------------------------------------------

Population error |

RMSEA | 0.053 Root mean squared error of approximation

90% CI, lower bound | 0.000

upper bound | 0.097

pclose | 0.418 Probability RMSEA <= 0.05

---------------------+------------------------------------------------------

Information criteria |

AIC | 9598.776 Akaike's information criterion

BIC | 9715.870 Bayesian information criterion

---------------------+------------------------------------------------------

Baseline comparison |

CFI | 0.974 Comparative fit index

TLI | 0.942 Tucker-Lewis index

---------------------+------------------------------------------------------

Size of residuals |

SRMR | 0.055 Standardized root mean squared residual

CD | 0.744 Coefficient of determination

----------------------------------------------------------------------------

Standardized results:

. sem (Mindfulness -> cent_awareness, ) (Mindfulness -> cent_nonreact, ) (Mindfulness -> cent_nonjudge, ) ///

> (Mindfulness -> cent_desposstep, ) (Mindfulness -> cent_desnegstep, ) (cent_desposstep -> cent_poc, ) ///

> (cent_desposstep -> cent_situ_conf, ) (cent_desposstep -> cent_reappraisal, ) (cent_desnegstep -> cent_poc, ) ///

> (cent_desnegstep -> cent_situ_conf, ) (cent_desnegstep -> cent_reappraisal, ) (cent_poc -> cent_pacs, ) ///

> (cent_situ_conf -> cent_pacs,) (cent_reappraisal -> cent_pacs, ) (Mindfulness -> cent_pacs, ) ///

> (cent_desnegstep -> cent_pacs, ) (cent_desposstep -> cent_pacs, ), cov(e.cent_poc*e.cent_reappraisal) ///

> cov(e.cent_nonreact*e.cent_desposstep) cov(e.cent_nonreact*e.cent_situ_conf) latent(Mindfulness ) standardized

Endogenous variables

Observed:     cent_desposstep cent_desnegstep cent_poc cent_situ_conf cent_reappraisal cent_pacs

Measurement:  cent_awareness cent_nonreact cent_nonjudge

Exogenous variables

Latent:       Mindfulness

Fitting target model:

Iteration 0:   log likelihood = -4824.9965  (not concave)

Iteration 1:   log likelihood = -4808.8774  (not concave)

Iteration 2:   log likelihood = -4778.0793  (not concave)

Iteration 3:   log likelihood = -4765.5123

Iteration 4:   log likelihood = -4764.2758

Iteration 5:   log likelihood = -4761.5824

Iteration 6:   log likelihood = -4761.3891

Iteration 7:   log likelihood = -4761.3881

Iteration 8:   log likelihood = -4761.3881

Structural equation model                       Number of obs     =        161

Estimation method  = ml

Log likelihood     = -4761.3881

( 1)  [cent_awareness]Mindfulness = 1

-------------------------------------------------------------------------------------------------------

                                      |                 OIM

                         Standardized |      Coef.   Std. Err.      z    P>|z|     [95% Conf. Interval]

--------------------------------------+----------------------------------------------------------------

Structural                            |

  cent_desposstep                     |

                          Mindfulness |   .4316191   .0804179     5.37   0.000     .2740029    .5892352

                                _cons |  -5.37e-09    .078811    -0.00   1.000    -.1544668    .1544668

  ------------------------------------+----------------------------------------------------------------

  cent_desnegstep                     |

                          Mindfulness |  -.6735287   .0666559   -10.10   0.000    -.8041719   -.5428855

                                _cons |   2.64e-09    .078811     0.00   1.000    -.1544668    .1544668

  ------------------------------------+----------------------------------------------------------------

  cent_poc                            |

                      cent_desposstep |   .2630245   .0786652     3.34   0.001     .1088436    .4172054

                      cent_desnegstep |   .1663648   .0802513     2.07   0.038     .0090752    .3236545

                                _cons |  -4.33e-09   .0759447    -0.00   1.000    -.1488488    .1488488

  ------------------------------------+----------------------------------------------------------------

  cent_situ_conf                      |

                      cent_desposstep |   .3507359   .0739116     4.75   0.000     .2058719    .4955999

                      cent_desnegstep |  -.0602825   .0775899    -0.78   0.437    -.2123559    .0917908

                                _cons |  -1.10e-08   .0731313    -0.00   1.000    -.1433347    .1433346

  ------------------------------------+----------------------------------------------------------------

  cent_reappraisal                    |

                      cent_desposstep |   .3081953   .0768178     4.01   0.000     .1576352    .4587554

                      cent_desnegstep |  -.0043783   .0804113    -0.05   0.957    -.1619816    .1532249

                                _cons |  -6.13e-09   .0749415    -0.00   1.000    -.1468826    .1468826

  ------------------------------------+----------------------------------------------------------------

  cent_pacs                           |

                      cent_desposstep |   .0226371   .0809549     0.28   0.780    -.1360315    .1813057

                      cent_desnegstep |   .1009671   .1080276     0.93   0.350    -.1107631    .3126972

                             cent_poc |    .083906   .0700693     1.20   0.231    -.0534272    .2212392

                       cent_situ_conf |  -.4370251   .0662433    -6.60   0.000    -.5668595   -.3071907

                     cent_reappraisal |  -.1395111   .0711713    -1.96   0.050    -.2790044   -.0000179

                          Mindfulness |  -.2018547   .1324634    -1.52   0.128    -.4614782    .0577688

                                _cons |  -8.07e-09   .0657776    -0.00   1.000    -.1289217    .1289217

--------------------------------------+----------------------------------------------------------------

Measurement                           |

  cent_awareness                      |

                          Mindfulness |   .6092498   .0674688     9.03   0.000     .4770134    .7414861

                                _cons |   6.19e-09    .078811     0.00   1.000    -.1544668    .1544668

  ------------------------------------+----------------------------------------------------------------

  cent_nonreact                       |

                          Mindfulness |   .3919424   .0812958     4.82   0.000     .2326056    .5512793

                                _cons |   2.61e-09    .078811     0.00   1.000    -.1544668    .1544668

  ------------------------------------+----------------------------------------------------------------

  cent_nonjudge                       |

                          Mindfulness |   .7235337   .0633178    11.43   0.000     .5994331    .8476344

                                _cons |   1.31e-08    .078811     0.00   1.000    -.1544668    .1544668

--------------------------------------+----------------------------------------------------------------

                 var(e.cent_awareness)|   .6288147   .0822107                      .4866731    .8124713

                  var(e.cent_nonreact)|   .8463811   .0637266                      .7302582    .9809694

                  var(e.cent_nonjudge)|   .4764989   .0916252                      .3268781    .6946052

                var(e.cent_desposstep)|    .813705   .0694198                      .6884117    .9618021

                var(e.cent_desnegstep)|   .5463591   .0897894                      .3959046    .7539903

                       var(e.cent_poc)|   .9285825   .0407913                      .8519781    1.012075

                 var(e.cent_situ_conf)|   .8610573   .0502034                      .7680744    .9652967

               var(e.cent_reappraisal)|   .9042119   .0441858                      .8216274    .9950973

                      var(e.cent_pacs)|    .655852    .062762                       .543688    .7911556

                      var(Mindfulness)|          1          .                             .           .

--------------------------------------+----------------------------------------------------------------

cov(e.cent_nonreact,e.cent_desposstep)|   .2744777   .0777877     3.53   0.000     .1220167    .4269388

cov(e.cent_nonreact,e.cent_situ_conf)|   .2231382   .0729899     3.06   0.002     .0800806    .3661958

    cov(e.cent_poc,e.cent_reappraisal)|   .2696886    .073079     3.69   0.000     .1264564    .4129207

-------------------------------------------------------------------------------------------------------

LR test of model vs. saturated: chi2(16)  =     23.16, Prob > chi2 = 0.1096
